# Ambient exposure to nitrogen dioxide and lung function: a multi-metric approach

**DOI:** 10.1007/s10661-025-13871-4

**Published:** 2025-03-20

**Authors:** Carmel Raz-Maman, Nili Borochov-Greenberg, Rafael Y. Lefkowitz, Boris A. Portnov

**Affiliations:** 1 School of Environmental Science, University of Haifa, 199 Aba Khushi Ave, Mt. Carmel, 3498838 Haifa, Israel; 2 Israel Defense Forces, Medical Corps, Tel Has Homer, Ramat Gan, Israel; 3 Department of Internal Medicine (Yale Occupational and Environmental Medicine Program), Yale School of Medicine, Yale University, New Haven, CT 06510 USA

**Keywords:** Exposure assessment, Exposure metrics, Nitrogen Dioxide, Air pollution, Lung function, Spatial interpolation

## Abstract

Most studies evaluating chronic ambient exposure to nitrogen dioxide (NO_2_) have used averages as the exclusive exposure metric. However, this approach may lead to an underestimation of potential health effects. The objective of this study is to evaluate the association between ambient exposure to NO_2_ assessed by various metrics, and lung function in a cohort of healthy male youth. A cross-sectional analysis of 5,462 subjects was conducted using multivariate linear regression. Exposure to NO₂ was assessed by spatial interpolation using Empirical Bayesian Kriging (EBK). Five different exposure metrics were evaluated over two years, including average concentration, the number and intensity of exceedances of the 24-h NO_2_ World Health Organization air quality guideline (AQG), and the number and intensity of 1-h NO_2_ peaks. Lung function indices, including percent predicted forced expiratory volume in one second (FEV_1_), forced vital capacity (FVC), forced expiratory flow between 25% and 75% of vital capacity (FEF_25-75_), and FEV_1_/FVC ratio, were assessed. The intensity of the 24-h AQG exceedances was associated with the largest reductions in FEV_1_ (-0.82%, 95% CI: -1.61%, -0.03%) and FVC (-1.03%, 95% CI: -1.86%, -0.20%), while FEF_25-75_ showed a significant decline only with the 1-h peak intensity metric (-2.78%, 95% CI: -5.02%, -0.54%). The study results support integrating diverse exposure metrics as part of NO_2_ chronic exposure assessment, as these metrics may capture a wider range of potential health effects that could be underestimated or overlooked when relying only on average concentrations.

## Introduction

Nitrogen dioxide (NO_2_) is an ambient pollutant primarily resulting from the combustion of fossil fuels by motor vehicles, power plants, and industry, to which individuals worldwide are commonly exposed (US EPA, 2016; WHO, 2021). Exposure to NO_2_ has been linked to a wide range of adverse health effects, including reduced lung function (LF), increased airway inflammation, elevated probability of developing chronic obstructive pulmonary diseases (COPD), and an increased likelihood of asthma exacerbations (Boogaard et al., 2022; Chen et al., 2022; Masroor et al., 2023; Pedersen et al., 2023). However, studies examining the association between long-term exposure to NO_2_ and LF have often demonstrated inconsistent results; some studies reported a significant decrease in LF (Gauderman et al., 2015; Knibbs et al., 2018; Tsui et al., 2018), while others did not (Chen et al., 2023; Hwang et al., 2015; Morales et al., 2015). A potential reason for these differences in findings might be attributed to the exposure assessment conducted to evaluate the effect of NO_2_ on LF. As reviewed by the US Environmental Protection Agency (US EPA, 2016), most of the long-term studies evaluating the association between exposure to NO_2_ and related health outcomes have utilized averages as the exclusive exposure parameter. Yet, considering the exposure profile (i.e., the temporal pattern in which exposure occurs) could reveal exposure-related health effects that may be underestimated or remain undetected when relying on the average as the exclusive exposure parameter (Berhane et al., 2004; Greenberg et al., 2017; Raz-Maman et al., 2024; US EPA, 1992; Virji & Kurth, 2021).

The current study aims to test this assumption by evaluating the effect of ambient exposure to NO_2_ assessed by Empirical Bayesian Kriging (EBK) using various exposure metrics over a two-year period on LF in a cohort of otherwise healthy male youth. The exposure metrics evaluated in this study included average concentration, the number and intensity of exceedances of the updated World Health Organization (WHO) 24-h NO_2_ air quality guideline (AQG) (WHO, 2021), and the number and intensity of 1-h NO_2_ peaks.

## Methods

### Study design and cohort

The current cross-sectional research evaluates the association between exposure to NO_2_, assessed by EBK using various metrics, and LF. The study population comprises healthy males aged 16–18 years, with complete medical background questionnaires and no physician-diagnosed asthma. Participants were assessed at the Israeli Naval Medical Institute (INMI) from 2012 to 2019 as part of routine medical screening for a voluntary special forces unit. The study's original cohort, before applying exclusion and inclusion criteria, was the same as in our previous research on ozone exposure and LF (Raz-Maman et al., 2024).

### Medical and demographic data

Information on participants' medical history (including asthma status) was drawn from general practitioner reports and military medical screening assessments, which were recorded in each subject's medical file. The smoking status of each individual was obtained from questionnaires. Current smokers were defined as those who reported smoking at the time of the survey, while recent smokers were defined as subjects who reported regularly smoking in the year before the spirometry date. Socioeconomic status was assessed by participants' place of residence, as classified by the Israeli Central Bureau of Statistics (ICBS, 2021). Detailed information on medical data collection and socioeconomic status classification can be found in our previous publication (Raz-Maman et al., 2024).

### Spirometry tests

Lung function testing was conducted by trained medical staff at the INMI who recorded height measurements and performed spirometry following the ATS/ERS 2005 protocol (Miller, 2005). Spirometry measured forced expiratory volume in one second (FEV₁), forced vital capacity (FVC), and forced expiratory flow between 25% and 75% of vital capacity (FEF₂₅₋₇₅). FEV₁ and FVC were derived from the best spirometry maneuver, defined as the trial yielding the highest sum of FEV₁ and FVC. The outcome variables included percent predicted FEV₁, percent predicted FVC, the FEV₁/FVC ratio, and percent predicted FEF₂₅₋₇₅. Predicted values for spirometry indices were calculated using reference equations from the Global Lung Function Initiative (GLI) (Quanjer et al., 2012). Additional details regarding the spirometry testing procedure and predicted values calculation used in this study can be found in our earlier publication (Raz-Maman et al., 2024).

### NO_2_ measurements and spatial analysis

NO_2_ levels were measured by 105 Ambient Air Quality Monitoring Station (AAQMS) using the chemiluminescence method according to the EN-14211 standard (EU, 2012; MoEP, 2022). This study utilized data from background NO_2_ AAQMS with at least 75% of daily measurements available during the two-year study period.

A two-year exposure period was selected as per a previous review of the literature and meta-analysis (Raz-Maman et al., 2022). NO_2_ measurements were extracted from AAQMS for the two years preceding the spirometry tests. Since participants underwent spirometry testing annually between the end of March and July, we extracted the measurements from AAQMS corresponding to the prior two-year period, encompassing the 24 months from April to March. For instance, for subjects examined in 2019, we extracted AAQMS measurements from April 2017 to March 2019. These measurements were then used to calculate exposure by different metrics, as detailed in the following section.

Empirical Bayesian Kriging (EBK), an advanced geostatistical interpolation method that automates model building for more accurate predictions (Gribov & Krivoruchko, 2020; Krivoruchko, 2012), was employed to produce predicted concentration surfaces based on the calculated exposure metrics derived from the extracted measurements. These predicted concentration surfaces were then used to estimate exposure at each participant's home address. The spatial resolution for the EBK geostatistical interpolations, represented by the cell size, was set at 225m^2^ (15 m x 15 m). Spatial analysis was conducted with ArcGIS Pro (Esri Inc., 2023).

### Exposure assessment metrics

Exposure was assessed across various metrics, including average, the number and intensity of exceedances of the WHO 24-h AQG, and the number and intensity of 1-h peaks. All metrics were calculated over the two-year study period preceding the spirometry test. The exposure assessment metrics in the current study are distinct from but share similar principles to those outlined in our previous publication (Raz-Maman et al., 2024).

The two-year average was determined by averaging the 24-h NO_2_ measurements over two years. To calculate the intensity of AQG exceedances, we averaged only the 24-h measurements that exceeded the 25 μg/m^3^ threshold, corresponding to the WHO 24-h AQG value (WHO, 2021). Additionally, we evaluated the total number of AQG exceedances by summing the number of the 24-h measurements that exceeded 25 μg/m^3^ over two years. For AAQMS with less than 100% 24-h measurements (75%−99%) over the two-year study period, we evaluated the number of missing AQG exceedances using the following calculation:

$$\frac{\textit{No. of AQG exceedances measured}}{\textit{No. of available daily measurements over two years}} \times \begin{array}{c}\textit{No. of missing daily measurements} \\\textit{\vspace{2pt} over two years}\end{array}$$

The calculated number of the missing AQG exceedances was added to the measured AQG exceedances to obtain the total number of AQG exceedances parameter.

The NO_2_ peak threshold was set at 50 μg/m^3^, representing 1/4 of both the former WHO 1-h NO_2_ AQG (WHO, 2006) and the current 1-h ambient value specified in Israel's clean air regulations of 200 μg/m^3^ (State of Israel, 2011). Peak intensity was calculated by averaging only the 1-h levels exceeding the peak threshold of 50 μg/m^3^ over the two-year study period. The total number of peaks was calculated by summing the number of the 1-h measurements exceeding the peak threshold. For AAQMS with less than 100% hourly measurements over the two-year study period, the number of missing peaks was evaluated using the following calculation:

$$\frac{\textit{No. of measured peaks}}{\textit{No. of available hour measurements over two years}} \times \begin{array}{c}\textit{No. of missing hour measurements} \\\textit{over two years}\end{array}$$

The missing peaks were combined with the measured peaks to establish the total number of peaks metric.

### Statistical analysis

We used descriptive statistics to determine the baseline characteristics of the study cohort and to describe multiple exposure metrics. Exposure metrics, including the average concentration, the number and intensity of exceedances of the WHO 24-h AQG, and the number and intensity of 1-h peaks, were analyzed as continuous variables. Similarly, percent predicted FEV_1_, percent predicted FVC, percent predicted FEF_25–75_, and the FEV_1_/FVC ratio were also assessed as continuous variables. Multivariate linear regression was used to assess the correlation between different NO_2_ exposure metrics and LF. Regression coefficients for the average concentration, AQG exceedances intensity, and peak intensity metrics were multiplied by 10 to indicate changes in LF per 10 μg/m^3^. Additionally, the coefficients for the total number of AQG exceedances were multiplied by 100 to represent 100 exceedances of the 24-h WHO AQG, and those for the total number of peaks were multiplied by 1000 to represent an increase of 1000 1-h peaks. Analyses were controlled for body mass index (BMI), obtained from participants' medical records, and were also adjusted for childhood asthma, smoking, and socioeconomic status. Statistical analyses were conducted by SPSS Statistics software version 27 (IBM Corp., 2020).

## Results

### Study cohort

A total of 6,013 adolescents underwent medical screening at the INMI over the study period (2012–2019), of whom 536 did not meet inclusion criteria due to incomplete medical background questionnaires. An additional 13 subjects diagnosed with asthma were excluded. Furthermore, 2 participants were excluded due to missing spirometry variables. Consequently, 5,462 participants were eligible for analysis. As mentioned in the methods section, our previous study on ozone utilized the same initial pool of 6,013 participants but examined only 665 participants from this cohort (Raz-Maman et al., 2024).

Table 1 provides the baseline characteristics of the study cohort. The mean age of the study participants was 17.1 years, with an average BMI of 22.3 kg/m^2^.

**Table 1:** Baseline characteristics of the study cohort

Variable	Study cohort (n = 5,462)
Age (years); mean ± SD	17.1 ± 0.3
BMI (kg/m^2^); mean ± SD	22.3 ± 2.5
Childhood asthma; number (%)	123 (2.3)
Current or recent smoker; number (%)	173 (3.2)
**Socioeconomic status (SES); number of subjects in the SES group (%):**	
• Low (1–4)	938 (17.2)
• Medium (5–7)	2,791 (51.1)
• High (8–10)	1,733 (31.7)
**LF indices; mean ± SD:**	
• FEV_1_ (% predicted)	97.81 ± 10.25
• FVC (% predicted)	96.29 ± 11.03
• FEV_1_/FVC (%)	87.51 ± 6.65
• FEF_25–75_ (% predicted)	99.74 ± 20.21

BMI—body mass index; SD—standard deviation; FEV_1_ -forced expiratory volume in one-second; FVC—forced vital capacity; FEV_1_/FVC—the ratio between FEV_1_ and FVC; FEF_25–75_—forced expiratory flow between 25% and 75% of vital capacity

### Distribution of exposures using various metrics

The distribution of exposures for the study population using various metrics including average concentration, the number and intensity of exceedances of the AQG, and the number and intensity of 1-h NO_2_ peaks is described in Table 2.

**Table 2:** NO_2_ exposure of study cohort (n = 5,462) evaluated by multiple metrics

Exposure metric	Mean	SD	Minimum	Maximum	Median	IQR
Average (μg/m^3^)	18.00	5.15	5.78	34.43	17.83	7.92
AQG exceedances intensity (μg/m^3^)	33.79	3.49	26.05	45.17	33.35	4.92
Total number of AQG exceedances	165	95	3	443	154	150
Peak intensity (μg/m^3^)	64.30	2.44	57.09	74.59	64.39	3.13
Total number of peaks	1,241	945	0	4,579	982	1,408

IQR- Inter Quartile Range; AQG- Air Quality Guideline

An average of 165 AQG exceedances (23% of the 730 days measured during the two-year study period) were estimated for the study cohort. Additionally, 1,241 1-h NO_2_ peaks were estimated, representing 7% of the 17,520 h measured during the two-year study period. The average concentration of AQG exceedances intensity was 33.79 μg/m^3^, and the average peak intensity was 64.30 μg/m^3^.

### *Association between NO*_*2*_* exposure metrics and LF indices*

**Fig. 1:**
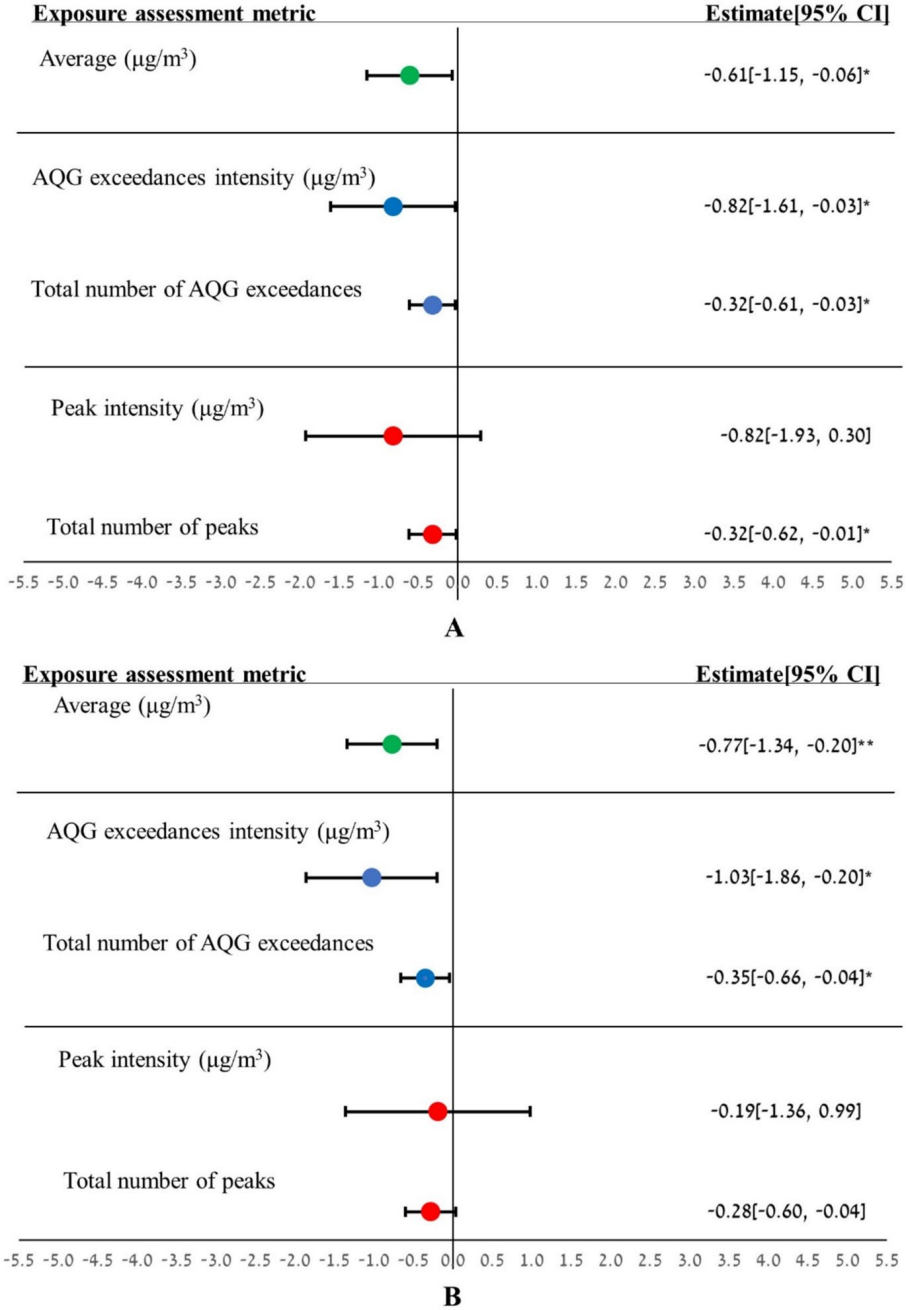

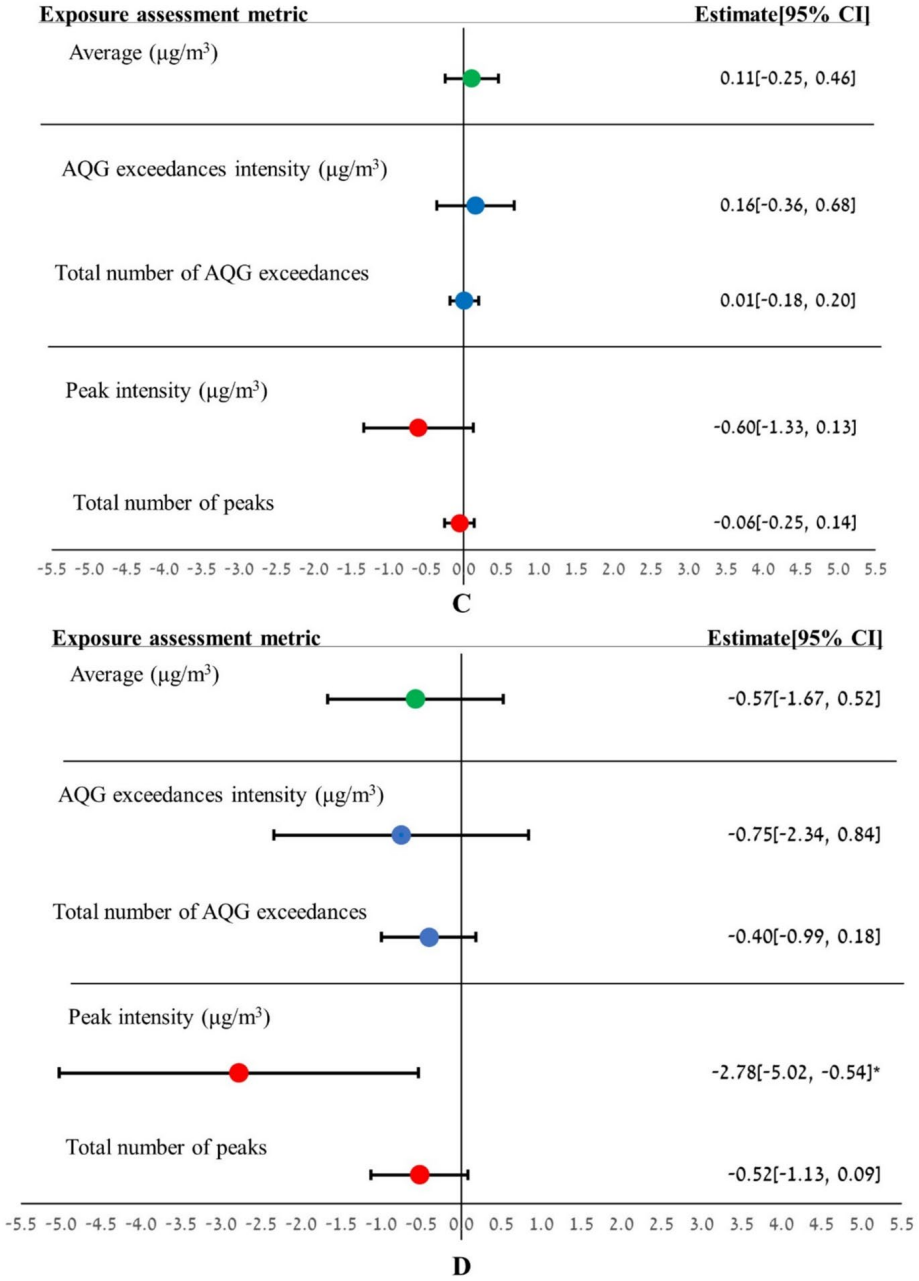
Estimates of differences in percent predicted FEV_1_ (**A**), percent predicted FVC (**B**), FEV_1_/FVC ratio in percentage (**C**), and percent predicted FEF_25–75_ (**D**) associated with NO_2_ exposure, assessed using various metrics. (Results demonstrate percent changes in LF per 10 μg/m^3^ increase for average, AQG exceedances intensity, and peak intensity metrics. For the total number of AQG exceedances, changes are per 100 AQG exceedances, and for the total number of peaks, per 1000 peaks. Analyses were adjusted for BMI, childhood asthma, current/recent smoking, and socioeconomic status. CI denotes confidence interval. *p < 0.05, **p < 0.01)

Figure 1 presents the results of the multivariate linear regression. Percent predicted FEV_1_, percent predicted FVC, and percent predicted FEF_25–75_ demonstrate significant decreases with increasing NO_2_ exposures, assessed using various metrics. The multivariate linear regression analysis found that a 10 μg/m^3^ increase in average NO_2_ concentration over the two years preceding the spirometry test was significantly correlated with a 0.61% decrease in FEV_1_ (95% CI: −1.15%, −0.06%) and a 0.77% decrease in FVC (95% CI: −1.34%, −0.20%). The largest decreases in FEV_1_ and FVC were detected when the AQG exceedances intensity metric was used, resulting in reductions of 0.82% (95% CI: −1.6%, −0.03%) and 1.03% (95% CI: −1.86%, −0.20%) per 10 μg/m^3^ NO_2_, respectively. A significant decrease in FEF_25–75_ was observed only with the peak intensity metric, showing a decline of 2.78% (95% CI: −5.02%, −0.54%) per 10 μg/m^3^ NO_2_, which represents the largest effect on LF indices. The total number of AQG exceedances was significantly correlated with a similar slight decline in FEV_1_ and FVC. For every increase of 100 AQG exceedances, there was a reduction of 0.32% (95% CI: −0.61%, −0.03%) in FEV_1_ and 0.35% (95% CI: −0.66%, −0.04%) in FVC. The same magnitude of effect was found for an increase of 1000 1-h peaks and FEV_1_, resulting in a decline of 0.32% (95% CI: −0.62%, −0.01%). We did not observe a significant correlation between exposure to NO_2_ assessed by different metrics and the FEV_1_/FVC ratio.

## Discussion

The study findings indicate that the two exposure assessment metrics representing the magnitude of high-level NO_2_ concentrations exceeding a defined threshold- AQG exceedances intensity and peak intensity, were associated with the most substantial declines in LF indices (FEV_1_, FVC, and FEF_25–75_). These results emphasize the importance of considering the pattern of exposure, including shorter-term but higher-intensity exposures, in long-term ambient exposure assessments.

Previous research (Liu et al., 2016; Ronaldson et al., 2022; Sun et al., 2018) has indicated that elevated levels of NO_2_ can induce respiratory morbidity and adverse health effects in a dose–response manner. Additionally, as outlined by Virji and Kurth (2021) and Albano et al. (2022), and noted in our previous publication (Raz-Maman et al., 2024), recurrent exposure to high levels of various air pollutants may overburden respiratory system immune defenses, thereby leading to respiratory morbidity. Consistent with this pathophysiological mechanism, our current study demonstrates that metrics accounting for exceedances of threshold limits might be more useful predictors of adverse health effects.

Table 3 presents a summary of studies from 2013 to 2023 with cohorts of over 300 subjects, examining the effects of long-term exposure (at least two months) to NO_2_ on LF indices: FEV_1_, FVC, FEV_1_/FVC ratio, and FEF_25–75_ (also referred to as Maximal Mid Expiratory Flow- MMEF). Of the studies outlined in Table 3, Tsui et al. (2018) utilized ordinary Kriging, a method sharing common principles with EBK (Mainuri & Owino, 2017; Wieskotten et al., 2023). Tsui et al. (2018) found results similar to ours, showing a significant reduction in FEV_1_ and FVC with NO_2_ exposures, and a non-significant effect on the FEV_1_/FVC ratio. As demonstrated in Table 3, all the studies utilized averages as the exposure parameter to assess the effect of NO_2_ on LF. Seven of these studies (Gauderman et al., 2015; Knibbs et al., 2018; Liu et al., 2020; Milanzi et al., 2018; Mölter et al., 2013; Tsui et al., 2018; Xing et al., 2020), exhibited a significant association with one or more LF indices analyzed. However, an equal number of studies did not reveal a significant correlation between prolonged exposure to NO_2_ and decreased LF (Barone-Adesi et al., 2015; Chen et al., 2015; Chen et al., 2023; Fuertes et al., 2015; Hwang et al., 2015; Morales et al., 2015; Urman et al., 2014). These varying outcomes may be due to relying solely on averages as the exposure metric, along with other potential limiting factors.

**Table 3:** studies examining the effect of long-term ambient exposure to NO_2_ on lung function in children and youth (2013 – 2023)

Author (year)	Study population (> 300)	Age range (years)	Exposure assessment method	Exposure assessment time frame (≥ 2 months)	NO_2_ increment	Effect of exposure to NO_2_ on FEV_1_, FVC, FEV_1_/FVC, FEF_25–75_
**Cross-sectional studies**						
Liu et al. (2020)	6,740	7–14	AAQMS combined with machine learning modeling	4 years average	7.3 μg/m^3^ (IQR)	FEV_1_ (ml): −93.18 (95%CI: −116.12, −70.24) FVC (ml): −123.27 (95%CI: −149.01, −95.54) No significant effect on FEF_25–75_
Xing et al. (2020)	6,740	7–14	Spatiotemporal model	4 years average	7.3 μg/m^3^ (IQR)	For the normal weight population: No effect on FEV_1_, FVC & FEF_25–75_. Overweight and obese populations showed higher OR for FEV_1_ < 85%, FVC < 85% & FEF_25–75_ < 85%
Knibbs et al. (2018)	2,630	7–11	AAQMS LUR	Lifetime, last year. lifetime without last year averages	4.03 ppb (IQR)	A significant decrease in percent predicted FEV_1_ and percent predicted FVC for all the periods analyzed. The largest decrease in pre-bronchodilator indices: 1.23% in percent predicted FEV_1_ &1.19% in percent predicted FVC. No significant effect on FEV_1_/FVC
Tsui et al. (2018)	1,016	6–15	Ordinary kriging	Lifetime, first year of life, 2–6 years of life averages	1 ppb	A significant increase in percent predicted FEV_1_ and percent predicted FVC for all the study periods analyzed (the largest effect is an increase of 0.43% for both percent predicted FEV_1_ & FVC), no significant effect on FEV_1_/FVC
Barone-Adesi et al. (2015)	4,884	9–10	DM	1 year average	4.9 μg/m^3^ (IQR)	No significant effect on FEV_1_ or FVC
Chen et al. (2015)	1,494	6–15	AAQMS	2 months average	6.02 ppb (IQR)	No significant effect on FEV_1_, FVC, FEV_1_/FVC ratio, and FEF_25–75_
Fuertes et al. (2015)	2,266	15	LUR	Annual averages of the year of birth, at 10- and 15-year	5.92 μg/m^3^ (IQR)	No significant effect on FEV_1_, FVC, and FEV_1_/FVC ratio
Morales et al. (2015)	620	4.5	LUR	First year of life, and recent year average	13.75 μg/m^3^, 25.41 μg/m^3^ (IQR)	No significant effect on FEV_1,_ FVC, and FEF_25–75_
Urman et al. (2014)	1,811	5–7	LUR	6 years average	19.4 μg/m^3^	No significant effect on FEV_1_ or FVC
**Longitudinal studies and combined cross-sectional and longitudinal studies**						
Chen et al. (2023)	4864	6–16	Space–time regression model	1 year average	5 μg/m^3^	No significant effect on FEV_1,_ FVC & FEF_25–75_
Milanzi et al. (2018)	915	8–16	LUR	4 years average	7.8 μg/m^3^	FEV_1_(% change): − 0.31% (95%CI: − 0.47, − 0.14). No significant effect on FVC
Hwang et al. (2015)	2,941	12	IDW	2 years average	7.73 ppb (IQR)	No significant effect on FEV_1_ and FVC
Gauderman et al. (2015)	1,585	11	AAQMS	4 years average	14.1 ppb	A decrease in NO_2_ was associated with a significant increase in FEV_1_ and FVC growth: FEV_1_ (ml): 91.4 (95%CI: 47.9, 134.9), FVC (ml): 168.9 (95%CI: 127.0, 210.7)
Mölter et al. (2013)	1,185	3–11	LUR	11 years average	1 μg/m^3^	For the longitudinal analysis: FEV_1_ (percent predicted): –0.83% (95%CI: –1.39, –0.28). No effect on FEV_1_ in the cross-sectional analysis

See footnote to Table 1; AAQMS—Ambient Air Quality Monitoring Station; DM- Dispersion Model; IDW- Inverse Distance Weighted; LUR- Land Used Regression model; IQR—Inter Quartile Range; OR—Odds Ratio. Studies exclusively assessing populations with morbidity are not included in the table

Our analyses demonstrated a significant association between exposure to NO_2_, assessed using multiple metrics, and a decrease in percent predicted FEV_1_ and percent predicted FVC, with the most substantial decrease observed in percent predicted FEF_25–75_. Although the study design does not allow for determining a causal relationship, a proposed interpretation may explain our results. As earlier noted (Raz-Maman et al., 2022), previous research suggests that NO_2_ exposure can induce oxidative stress, reducing lung tissue antioxidant defenses, leading to tissue injury, inflammation, and extracellular damage along the airways, potentially causing edema and fibrosis (Albano et al., 2022; Persinger et al., 2002; Petit et al., 2017), which may lead to the observed decline in FEV_1_, FVC, and FEF_25–75_. The effect observed in FEF_25–75_, a marker of small airways obstruction (Gold & Koth, 2016; Konstantinos Katsoulis et al., 2016), may be attributed to the deposition of NO_2_ in the small airways (Amaducci & Downs, 2024; ATSDR, 2024; Frampton et al., 2002). The significant association observed exclusively between the peak intensity metric and FEF_25–75_ could be particularly important, as a decline in FEF_25–75_ has been found to predict the development of COPD in subjects with normal LF (Kwon et al., 2020), and has also been identified as a predictor of airway hyperresponsiveness (Kim et al., 2021; Simon et al., 2010).

The current study findings demonstrate that the exposure metrics accounting for high levels of ambient NO_2_ (AQG exceedances intensity and peak intensity) as part of chronic exposure assessment, predict a greater decline in LF indices (FEV_1_, FVC, and FEF_25–75_), compared with the commonly used average exposure. In our previous study (Raz-Maman et al., 2024), which evaluated exposure to ambient ozone on LF using multiple metrics, we also found that the peak intensity metric showed the most substantial decline, albeit for a different LF parameter (FEV_1_/FVC ratio).

The present study has several strengths and limitations due to its methodology and study design, as emphasized in our earlier publication (Raz-Maman et al., 2024). Key strengths of our study include the incorporation of a comprehensive baseline medical history for each participant, including physician-diagnosed asthma. This enabled the exclusion of individuals with asthma, mitigating potential bias. Another strength of the study was the participants' high motivation to achieve the best spirometry results, as spirometry testing was part of medical screening for a voluntary special forces unit. This is particularly important, as spirometry relies significantly on the appropriate performance of the respiratory maneuvers. Additionally, the spirometry tests were conducted using the identical spirometer and in the identical clinical setting, thus maximizing consistency in lung function testing. A further strength of this study is the comprehensiveness of the AAQMS measurements used for the EBK spatial interpolation, with 72% of the AAQMS providing more than 90% of daily measurements during the two-year study period. However, the study also has certain limitations. The primary limitation of this study is the inability to assess personal exposure, as the exposure was estimated by EBK spatial interpolation. Moreover, our analysis did not control for additional pollutants. Furthermore, this study examined multiple exposure assessment methods and lung function indices, which may have introduced a potential risk of Type I error. Another possible limitation is the lack of control for physical activity and other potential confounders. Additionally, the current study focused on a single threshold level for NO₂ peaks. To gain a more comprehensive understanding of peak intensity effects, further research should examine a range of alternative threshold levels. Lastly, our study comprised healthy adolescents who performed spirometry as part of recruitment to a special forces unit; although the homogeneity of this cohort can also be viewed as a potential strength by decreasing the probability of hypothetical unaccounted influencing factors, it also restricts the study’s generalizability.

## Conclusion

This study demonstrates that incorporating additional parameters of the exposure profile as part of the chronic exposure assessment of ambient NO_2_ may reveal health effects that could otherwise be underestimated or remain undetected. Additional studies with more varied populations and exposure assessment methodologies may further validate our findings and present a more comprehensive depiction of how shorter-term exposure patterns impact LF and respiratory morbidity.

## Data Availability

The datasets generated during and/or analyzed during the current study is available from the corresponding author upon reasonable request.
